# Effect of type of anticoagulant, transportation time, and glucose in the culture media on neutrophil viability and function test results in dairy cattle

**DOI:** 10.1371/journal.pone.0311742

**Published:** 2024-10-10

**Authors:** Sanjana Malledevarahalli Chandrappa, Lei Xie, Sebastian Gonzalez Andueza, Hafez Sadeghi, Muhammad Hussnain Rashid, Mehrnaz Niazi, Kaixi Qiao, Qiang Dong, Leila Vincenti, Alessandro Ricci, Osvaldo Bogado Pascottini, Geert Opsomer

**Affiliations:** 1 Faculty of Veterinary Medicine, Department of Internal Medicine, Reproduction and Population Medicine, Ghent University, Merelbeke, Ghent, Belgium; 2 Department of Veterinary Sciences, University of Turin, Grugliasco, Turin, Italy; 3 College of Veterinary Medicine, Oklahoma State University, Stillwater, OK, United States of America; 4 College of Veterinary Medicine, Northwest A&F University, Yangling, Shaanxi, China; 5 School of Veterinary Medicine, University College Dublin, Belfield, Dublin 4, Ireland; Universidad Nacional de Colombia Campus Palmira, COLOMBIA

## Abstract

In dairy cattle research, *in vitro* assessment of innate immune function is commonly evaluated by flow cytometry via the quantitative analysis of circulating polymorphonuclear leukocytes (PMN) functionalities specifically focusing on the capacities for phagocytosis (PC) and oxidative burst (OB). Variations in these PMN functions, however, may not only be influenced by the health status of the animals but also by technical, non-animal related factors. Our objectives were to assess the PMN viability, PC and OB capacities from blood samples collected in tubes coated with different anticoagulants (acid citrate dextrose (ACD) and ethylenediaminetetraacetic acid (EDTA)) and stored for 0, 3, 6, 9, and 12 h at 4°C (to mimic transportation timeframe). Furthermore, we evaluated the PMN functionalities (PC and OB) in samples incubated in culture medium with glucose (7.2 mM) versus no glucose. Over five replicates, coccygeal blood samples were collected from three nulliparous Holstein heifers (5 ACD and 5 EDTA per heifer) and allocated in a refrigerated container (4°C) for 0, 3, 6, 9, and 12 h. At each time point, PMN were isolated using gradient centrifugation. Immunolabeled PMN (CH138A) were subjected to a tricolor fluorescent staining to evaluate their viability (viable, apoptotic, and necrotic PMN). Phagocytosis and OB were assessed by incubating PMN with fluorescent beads and by phorbol 12-myristate 13-acetate stimulation, respectively. The effects of anticoagulant type, storage time, and presence of glucose in the culture medium on PMN viability and function parameters were fitted in mixed linear regression models. The proportion of viable PMN at 0 h was similar for ACD and EDTA (92 ± 4.6% and 93 ± 4.6%, respectively) but it decreased to 78 ± 4.6% for ACD and 79 ± 4.6% for EDTA after 6 h of storage. The proportion of viable PMN was not different between ACD and EDTA at any time point. The proportion of PMN that engulfed beads (PC percentage) and the PC median fluorescence intensity (MFI) reached their highest value after 3 h of storage compared with the other time points. However, the anticoagulant type (ACD versus EDTA) and the presence of glucose in the culture medium did not influence these PC parameters. Oxidative burst MFI was higher in PMN incubated in glucose-supplemented culture medium versus no glucose. We demonstrated that technical factors interfere with the evaluation of PMN viability and functionality, which can potentially lead to bias in the findings of a research hypothesis. To conclude, the present study showed that the optimal timeframe for performing PMN function analyses is within 3 hours after blood sampling. Furthermore, the presence of 7.2 mM glucose in the culture medium, a common concentration in formulation of cell culture medium, increases the *in vitro* OB capacity, potentially masking any impairments in *in vivo* PMN dysfunctionality.

## Introduction

Polymorphonuclear leukocytes (PMN) are of paramount significance within the innate immune system, playing a pivotal role in the primary defence of dairy cattle against microbial pathogens [1,2]. Upon immune activation, PMN promptly migrate to targeted sites where they perform essential functions. Among these functions, phagocytosis (PC) and oxidative burst (OB) are the most commonly assessed in dairy cattle research using flow cytometry [3,4], offering valuable insights into the innate immune status at a given time point.

Multiple studies have demonstrated an association between innate immune function and diverse aspects of cattle health including colostrum consumption in newborn calves [5,6], metabolic stress in transition cows [7,8], and infectious diseases in postpartum cows [9,10]. However, since approximately 40 years ago, when Saad and Hageltorn (1985) [11] pioneered the use of flow cytometry in cattle health to evaluate PMN PC *in vitro* by incubation with fluorescent-labeled bacteria, the optimization and exploration of various protocols has broadened the range of parameters that can be evaluated through flow cytometry. Yet, to date, to the best of our knowledge, there exists no standardized protocol for measuring PMN viability and function via flow cytometry in dairy cattle.

The entire process starting from blood collection, conditions during sample transportation, PMN isolation, and culture settings, carries the potential to introduce bias into the experimental outcome. This bias may arise from its impact on both the viability and functionality of PMN. For blood collection, acid citrate dextrose (ACD) is a frequently employed anticoagulant. However, ACD includes dextrose at a concentration of 24.5g/L in its composition, thereby supplying an additional source of energy to PMN, which may eventually mask *in vivo* function deficiencies. Furthermore, chelating activities of distinct calcium complexing agents in ACD as well as in the anticoagulant ethylenediaminetetraacetic acid (EDTA), was also shown to potentially interfere with PMN functions [12,13]. Prolonged transportation time is another important factor that may extrinsically affect the evaluation of PMN viability and functional capacities. Prolonged blood storage can initiate apoptosis and necrosis pathways in PMN, leading to a decrease in their viability [14]. High proportions of necrotic PMN were shown to interfere with its fluorescence intensity when performing flow cytometry [7]. Regarding the *in vitro* culture conditions, most culture medium formulations and supplements are designed for specific objectives [15]. Particularly, the glucose concentration which is an essential energy source for cultured cells [16], exhibits significant variability (ranging from 5.5 to 55 mM) across commercially available culture media [17].

As mentioned above, some lab-related factors may influence with the *in vitro* assessment of PMN viability and functionality via flow cytometry. Thus, we hypothesized that the type of anticoagulant used for blood collection, transportation time, and the presence of glucose in the culture medium influence the viability (viable, apoptotic, and necrotic), PC and OB capacities of PMN *in vitro*. Our principal objective was to compare the viability and functionality of circulating PMN collected using ACD or EDTA tubes and stored for different times (0, 3, 6, 9, and 12 h) at 4°C to mimic the transportation timeframe. Furthermore, we investigated the effect of the glucose concentration (7.2 mM versus no glucose) in the culture medium on *in vitro* PMN PC and OB capacities.

## Material and methods

### Ethics declaration

Animal handling and sampling were approved by the Ethical Committee of Animal Testing of the University of Antwerp (Antwerp, Belgium) and the Flemish Government, Department of Strategy, International Policy, and Animal Welfare (Brussels, Belgium) with ECD-dossier 2019–44.

### Study design, sample collection, and PMN isolation

**Fig 1:**
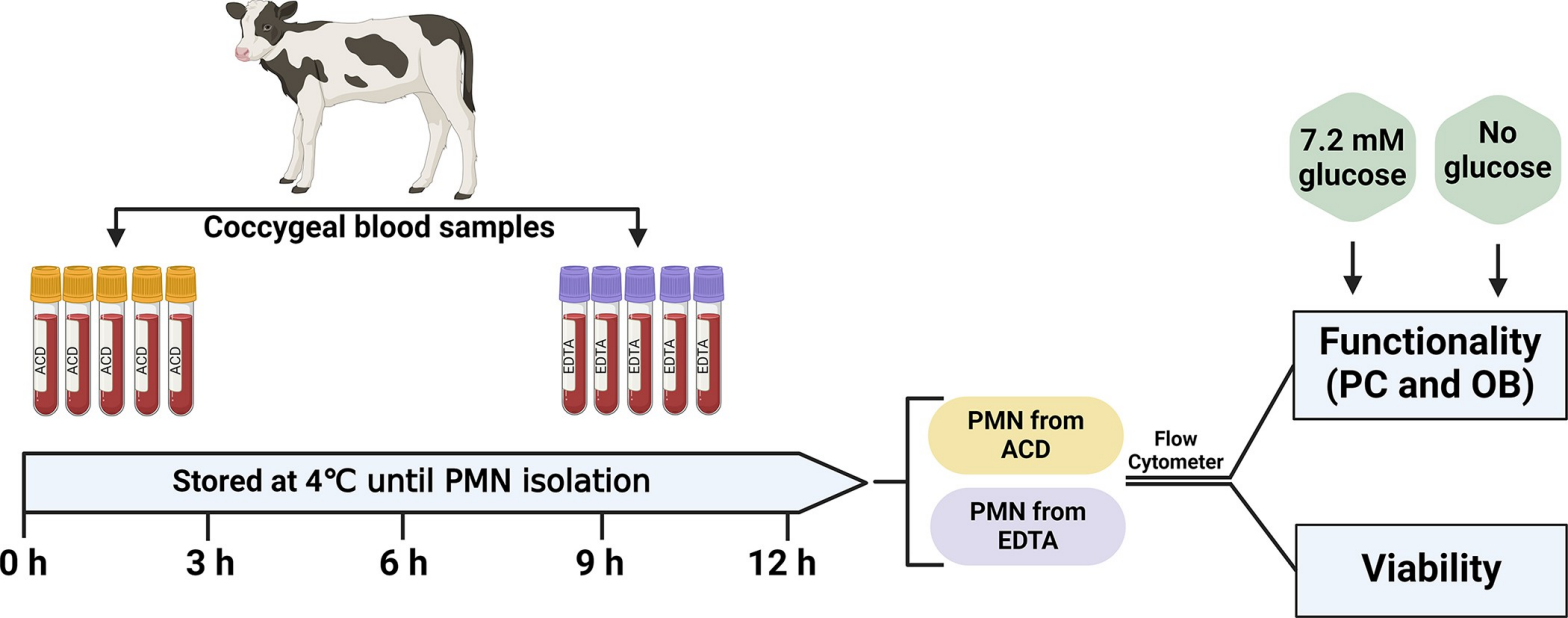
Schematic overview of the experimental design. Across five replicates, blood samples were collected from nulliparous Holstein heifers (n = 3) in acid citrate dextrose (ACD) and ethylene diamine tetra acetate (EDTA) coated tubes. Samples were stored at 4°C for 0, 3, 6, 9, and 12 h to mimic transportation timeframe. At each time point, polymorphonuclear leukocyte (PMN) were isolated to perform viability (viable, apoptotic, and necrotic PMN) and functionality (phagocytosis (PC) and oxidative burst (OB)) tests via flow cytometry. The functionality tests were performed in culture medium supplemented with 7.2 mM glucose and without glucose supplementation.

The experimental design of the present study is shown in **Fig 1**. Nulliparous Holstein heifers (n = 3) from a commercial dairy farm (Nevele, East Flanders, Belgium) were housed in a free stall barn and received a total mixed ration and had *ad libitum* access to fresh water. At the time of sampling (September to October 2021), heifers were between 10 and 12 months of age and clinically healthy. Coccygeal blood samples were collected using 18-gauge needles into two types of anticoagulant vacuum tubes, ACD and EDTA. The ACD tubes (BD Vacutainer^®^, Becton Dickinson and Company, Plymouth, UK) contained 1.5 mL of trisodium citrate (22.0 g/L), citric acid (8.0 g/L), and dextrose (24.5 g/L), whereas the EDTA tubes (BD Vacutainer^®^, Becton Dickinson and Company, Plymouth, UK) contained 1.8 mg/mL potassium salt (K_2_E) EDTA solution. The blood samples were collected once per week for five consecutive weeks (n = 5 replicates) between 0700 and 0800 h (before feeding). At each replicate, each heifer contributed five ACD and five EDTA tubes, which were collected in one shot from the same blood vessel with the same needle. After blood collection, the tubes were mixed gently and immediately stored in a refrigerated container at 4°C. Within 30 min of blood collection, PMN isolation started from one set of samples (one ACD and one EDTA tube per heifer), which corresponds to the baseline sample (referred to as 0 h collection time). Subsequently, after 3, 6, 9, and 12 h of sampling a set of tubes (one ACD and one EDTA per heifer) was used for PMN isolation. For the sample size calculation, the unit of interest was the number of samples taken across the replicates (3 heifers and 5 replicates; n = 15 samples). This sample size is sufficient to detect differences (significance level = 0.05 and power = 80%) of 5 ± 4 (mean ± SD) in the percentage PMN performing PC when the culture medium is supplemented with or without glucose as shown by Garcia et al [18].

The PMN isolation procedure was conducted following the methodology as described by Lietaer et al. (2021). Briefly, 8 mL of blood from each tube was gently mixed with 20 mL of 1x phosphate buffered saline (PBS; Gibco/Thermo Fisher Scientific, Waltham, MA, USA) supplemented with 0.5 mM of EDTA disodium salt (Sigma-Aldrich, Oakville, ON, Canada). The blood mixture was then slowly overlayed on 8 mL of endotoxin-free Ficoll-Paque^TM^ PLUS (GE HealthCare, Uppsala, Sweden) and centrifuged at 340x g for 30 min at room temperature (RT). This centrifugation process resulted in the separation of the blood into three distinct layers, with the top plasma and buffy coat layers being discarded. Then, the bottom layer was kept, and 30 mL of distilled water (B. Braun, Melsungen, Germany) was added into it and mixed for 45 seconds, followed by the addition of 15 mL of 3x PBS to balance the osmotic pressure. Subsequently, the mixture underwent centrifugation at 220x g for 10 min at 4°C. After centrifugation, the supernatant was removed, and the resultant pellet was washed with 500 μL of 1x PBS. Then, the previously mentioned steps were repeated resulting in a clean pellet of PMN free of erythrocytes. This pellet was suspended in 1 mL of Dulbecco’s Modified Eagle Medium (DMEM) free of glucose and glutamine (Biological industries/Sartorius, Beit Haemek, Israel), supplemented with 0.1% bovine serum albumin (BSA, ≥ 96%, Sigma-Aldrich, Oakville, ON, Canada) and kept on ice. In the meanwhile, the PMN concentration was assessed in a Bürker chamber, and the PMN suspension was adjusted to 5 x 10^6^ of PMN/mL in two different tubes containing DMEM-BSA with no glucose nor glutamine and DMEM-BSA supplemented with 7.2 mM glucose (without glutamine). Cytospin slides were prepared and stained with Wright’s stain following the isolation of each sample. Briefly, 100 uL of cells suspension was added to the cytoclip set, which included filter paper and a microscope slide. The assembly was then centrifuged, allowing the cells to adhere to the slide. After centrifugation, the cells were stained using Wright’s stain to assess morphology and purity. Cells were assessed for purity, and only samples with greater than 80% PMN were further processed.

### Viability test: Viable, apoptotic and necrotic PMN

Viability experiments were run in duplicate. To do so, 1 × 10^6^ PMN were allocated in 1.5 mL microcentrifuge tubes and spined at 376x g for 5 min at 4°C. Pelleted PMN were then suspended in 100 μL flow cytometry staining buffer (FACS buffer; 1x PBS with 1% BSA and 0.073% EDTA) supplemented with 1 μL anti-bovine granulocyte antibody IgM “CH138A” (BOV2067, WSU Pullman campus, USA) for 30 min at 4°C in the dark. Following CH138A antibody incubation, the PMN were washed with 200 μL FACS buffer (spined at 376x g for 5 min at 4°C) and then incubated with 100 μL FACS buffer with goat anti-mouse IgM (final concentration was 4.80 g/mL) for 15 min at 4°C in the dark. After incubation, PMN were washed in FACS buffer (200 μL, 376x g, 5 min, 4°C). In the meanwhile, 1 mL of Ca^2+^-rich incubation buffer (10 mM HEPES, 140 mM NaCl, 5 mM CaCl_2_, pH 7.4) was prepared and mixed with 20 μL of Annexin-V-FLUOS (fluorescein; Roche Diagnostics GmbH, Mannheim, Germany) and 20 μL of propidium iodide (PI; final concentration of 1 μg/mL, Molecular Probes, Invitrogen, Belgium). Lastly, 100 μL of the Annexin-PI solution was added to the pelleted PMN and incubated at RT for 10 min in the dark. Autofluorescence controls were also included alongside (equivalent quantity of PMN without any fluorescent markers), to ensure proper compensation and accurate interpretation of the flow cytometry results. All samples were put on ice and protected from light until flow cytometric analysis.

### Functionality tests: Phagocytosis and oxidative burst

PMN functionality tests (PC and OB) were conducted as described by Lietaer et al. (2021). All tests were run in duplicate both in DMEM-BSA without glucose and glutamine and in DMEM-BSA without glutamine but supplemented with 7.2 mM glucose.

For the PC capacity assessment, 100 mg Zymosan-A derived from *Saccharomyces cerevisiae* (2149, Sigma-Aldrich, Winston Park Dr, Oakville, ON, Canada) was added to 10 mL serum derived from healthy dairy cows and gently agitated for 1 h at 38.5°C to obtain zymosan-activated serum (ZAS). This was centrifuged at 390x g for 10 min to remove the precipitate, after which the supernatant was aliquoted and stored at -20°C. On the day of the experiment, 50 μL of ZAS was mixed with 200 μL DMEM-BSA medium (with and without glucose) containing 1 × 10^6^ PMN in 1.5 mL microcentrifuge tubes. Also, 1 μL of fluorescently labeled beads (FluoSpheres carboxylate, yellow-green (505/515), Life Technologies Corp., Eugene, OR, USA) was added into the microcentrifuge tubes. This mixture was incubated for 30 min at 38.5°C in the dark under gently agitation. After incubation, PMN were washed in FACS buffer (200 μL, 376x g, 5 min, 10°C). The PMN were then resuspended in 200 μL FACS buffer and preserved on ice under light-protected conditions until analysis. To compensate for the background fluorescence emitted by the PMN or ZAS, autofluorescence controls with no fluorescent beads added were also prepared alongside.

For the OB activity assessment, 2 μL of 2’,7’- dichlorodihydrofluorescein diacetate (H_2_DCFDA) (Life Technologies Corp., 29851 Willow Creek Rd, Eugene, OR 97402, USA) was added to a 200 μL suspension containing 1 × 10^6^ PMN in DMEM-BSA medium (with and without glucose) in 1.5 mL microcentrifuge tubes. The initial incubation was carried out for 15 min at 38.5°C in the dark. The PMN were subsequently stimulated with 200 μL phorbol 12-myristate 13-acetate (PMA; Sigma-Aldrich, 2149 Winston Park, Oakville, ON, Canada) at a final concentration of 200 ng/mL. Autofluorescence controls without PMA stimulation were included alongside. Following stimulation, the PMN were incubated for 15 min at 38.5°C in the dark. Then the PMN were washed in FACS buffer (200 μL, 376x g, 5 min, 4°C). The PMN were then resuspended in 200 μL FCAS buffer and preserved on ice under light-protected conditions until analysis.

### Flow cytometric analysis of PMN viability and functionality tests

The samples were analysed using a CytoFLEX 3-laser flow cytometer (Beckman Coulter Life Sciences Headquarters 5350, Indianapolis, Indiana 46268, USA). For each sample, 10,000 events were recorded, which were gated based on higher forward scatter (FSC) and side scatter (SSC) values on FSC vs. SSC plots. The acquisition velocity was 30–60 μL/min. Cells were measured for fluorescence at two wavelengths (488 and 638 nm), with the emitted fluorescence signals detected using specific emission filters (detailed information of the used filters can be found in **S1 Table**). CytExpert software (version 2.0.0.153, Beckman Coulter Diagnostics, Brea, California, United States) was used for data processing, and compensation was applied for multicolour configurations.

Viability analysis included all the events, with only the debris fraction identified on the FSC vs. SSC plot being excluded (PMN were identified based on the CH138A labelling). Three populations were differentiated based on viability staining: viable CH138A^+^ PMN with Annexin^-^/PI^-^, apoptotic CH138A^+^ PMN with Annexin^+^/PI^-^, and necrotic CH138A^+^ PMN with Annexin^+^/PI^+^.

For the PC and OB activity tests, the PMN population was identified and gated based on the FSC-A vs. FSC-H scatter plot, with aggregates removed. Fluorescence emitted in the assays was measured with the 525 ± 20 nm filter [fluorescein isothiocyanate (FITC) channel]. The percentage of PMN that performed PC was assessed. The MFI was determined for positive samples in both PC and OB assays. Moreover, the difference in MFI between positive samples and the relevant auto-fluorescent control (non-stained PMN) was determined for each parameter

### Statistical analyses

Collected data were exported and merged into a Microsoft Excel (Microsoft Corp., Redmond, WA, US) spreadsheet, where initial data exploration and organization were done using the PivotTables function (Microsoft Excel). The statistical analyses were performed using R-Studio (version 4.0.4; R Core Team, Vienna, Austria). PMN viability and function parameters were firstly analysed by Shapiro-Wilk’s test and when not normally distributed (*P* < 0.05), they were transformed (square root or log10). The function *lmer* of the R package *lme4* was used to fit mixed linear regression models. Storage time (0, 3, 6, 9, and 12 h), anticoagulant in the blood collection tube (ACD and EDTA), and their interaction were forced into each model to test their effects on PMN viability parameters (viable, apoptotic, and necrotic PMN). For PMN function parameters (PC and OB), the effect of glucose in the PMN function medium (glucose yes versus no) was also included in the mixed linear regression models independently and as a second-degree interaction with storage time and anticoagulant in the collection tube. All models accounted for the random effect of the replicate within the animal ID. Model residuals were assessed using a scatterplot of the studentized residuals for homoscedasticity, linear predictor for linearity, and a Shapiro-Wilk test for normality. For all transformed and non-transformed variables, the residuals of the models were normally distributed (Shapiro-Wilk’s *P* > 0.05). The denominator degrees of freedom were calculated with the Kenward-Roger method. Differences between levels of explanatory variables were assessed with Tukey’s post hoc test. Results are expressed as least squares means ± standard errors. The significance levels were set at *P* < 0.05.

## Results

**Fig 2:**
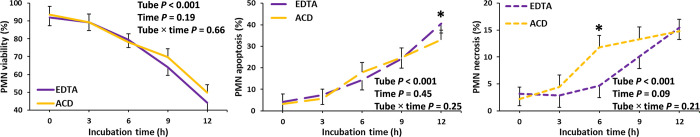
Flow cytometric evaluation of circulating polymorphonuclear leukocyte (PMN) viability (viable, apoptotic, and necrotic PMN). PMN were isolated from nulliparous Holstein heifers (n = 3) across five replicates. Linear regression models were fitted to evaluate the effect of storage time (0, 3, 6, 9 and 12 h), anticoagulant in the blood collection tube (acid citrate dextrose (ACD) and ethylenediaminetetraacetic acid (EDTA)), and their interaction on PMN viability parameters. Asterisks represent *P* < 0.05 between time point. Values are expressed as least square means ± standard errors.

Each of the PMN viability parameters changed from 0 to 12 h storage (*P* < 0.001; **Fig 2**). The PMN viability was similar (*P* > 0.98) at 0 h between ACD (92.1 ± 4.62%) and EDTA (93.6 ± 4.62%), and also at 3 h storage (89.3 ± 4.6% for both). However, the PMN viability decreased to 78.5 ± 4.62% for ACD and 79.6 ± 4.62% for EDTA after 6 h storage (*P* < 0.02). The PMN viability was not different (*P* = 0.66) between ACD and EDTA at any time point. Apoptosis increased (*P* < 0.05) from 3.34 ± 4.43% for ACD and 4.12 ± 4.43% for EDTA at 0 h storage to 7.95 ± 4.43% for ACD and 14.22 ± 4.43% for EDTA after 6 h storage. Interestingly, apoptosis was lower (*P* < 0.05) in ACD (32.97 ± 4.43%) than EDTA (40.58 ± 4.43%) at 12 h storage. For EDTA coated tubes, PMN necrosis was similar (*P* = 0.90) at 0 (3.21 ± 2.24%) and 6 h (4.70 ± 2.24%) but increased (*P* = 0.02) to (10.08 ± 2.24%) after 9 h storage. For ACD coated tubes, storage stimulated PMN necrosis from 2.19 ± 2.24% at 0 h to 11.79 ± 2.24% after 6 h (*P* = 0.02). The PMN necrosis was higher (*P* = 0.05) in ACD than EDTA after 6 h storage.

The effects of storage time, anticoagulant in the blood collection tube, and glucose in the culture medium on PC and OB are shown in **Tables 1** and **2**, respectively. Overall (ACD and EDTA tubes taken together), the PC percentage and PC MFI reached their highest point after 3 h of storage compared to the other storage time points (*P* = 0.003 and *P* = 0.02, respectively; **Table 1**). The type of anticoagulant and glucose supplementation did not affect the PC percentage nor the PC MFI (*P* > 0.23). Overall (ACD and EDTA tubes taken together), the OB MFI was higher at 0 and 3 h of storage compared to the other storage time points (*P* < 0.05). Interestingly, the OB MFI was greater when it was performed in glucose supplemented culture medium versus no glucose (*P* < 0.001), particularly within the first 6 h of storage (**Table 2**).

**Table 1:** Flow cytometric evaluation of circulating polymorphonuclear leukocyte (PMN) phagocytosis percentage and median fluorescence intensity (MFI) isolated from nulliparous Holstein heifers (n = 3) across five replicates.

Storage time	Phagocytosis percentage				Phagocytosis MFI			
	ACD		EDTA		ACD		EDTA	
	Glucose	No glucose	Glucose	No glucose	Glucose	No glucose	Glucose	No glucose
**0 h**	47.1 ± 1.83	46.4 ± 1.83	45.9 ± 1.83	46.8 ± 1.83	238 ± 49.7	255 ± 49.7	229 ± 49.7	224 ± 49.7
**3 h**	51.9 ± 1.71	52.9 ± 1.71	49.9 ± 1.71	50.3 ± 1.71	356 ± 53.7	382 ± 55.2	356 ± 53.7	302 ± 53.7
**6 h**	50.0 ± 2.26	50.1 ± 2.26	48.5 ± 2.26	46.9 ± 2.26	322 ± 53.1	316 ± 53.6	264 ± 53.1	224 ± 53.1
**9 h**	47.0 ± 2.47	47.4 ± 2.47	48.3 ± 2.47	47.3 ± 2.47	209 ± 59.1	238 ± 59.1	266 ± 59.1	253 ± 59.1
**12 h**	46.9 ± 1.68	48.8 ± 1.68	46.8 ± 1.68	48.2 ± 1.68	242 ± 43.6	288 ± 43.6	263 ± 43.6	286 ± 43.6

Linear regression models were fitted to evaluate the effect of storage time (0, 3, 6, 9 and 12 h; *P* = 0.03), anticoagulant in the blood collection tube (acid citrate dextrose (ACD) and ethylenediaminetetraacetic acid (EDTA); *P* = 0.23), and presence of glucose in the culture medium (7.2 mM glucose and no glucose; *P* = 0.75) on PMN phagocytosis percentage and MFI. Values are expressed as least square means ± standard errors. Median fluorescence intensity data were log^10^ transformed to achieve normalization.

**Table 2:** Flow cytometric evaluation of circulating polymorphonuclear leukocyte (PMN) oxidative burst median fluorescence intensity (MFI) isolated from nulliparous Holstein heifers (n = 3) across five replicates.

Storage time	ACD		EDTA	
	Glucose	No glucose	Glucose	No glucose
**0 h**	4.01 ± 0.08^b^	3.71 ± 0.08^a^	3.93 ± 0.08^ab^	3.71 ± 0.08^a^
**3 h**	4.03 ± 0.08^ab^	3.66 ± 0.08^a^	4.06 ± 0.08^ab^	3.82 ± 0.09^a^
**6 h**	3.80 ± 0.10^b^	3.41 ± 0.10^a^	3.68 ± 0.10^ab^	3.45 ± 0.10^a^
**9 h**	3.88 ± 0.14	3.64 ± 0.16	3.69 ± 0.14	3.56 ± 0.15
**12 h**	3.90 ± 0.11	3.73 ± 0.12	3.80 ± 0.12	3.51 ± 0.12

Linear regression models were fitted to evaluate the effect of storage time (0, 3, 6, 9 and 12 h; *P* < 0.001), anticoagulant in the blood collection tube (acid citrate dextrose (ACD) and ethylenediaminetetraacetic acid (EDTA); *P* = 0.16), and presence of glucose in the culture medium (7.2 mM glucose and no glucose; *P* < 0.001) on PMN oxidative burst MFI. Different superscripts within rows (^a^ and ^b^) represent *P* < 0.05. Values are expressed as least square means ± standard errors. Data were log^10^ transformed to achieve normalization.

## Discussion

We investigated the effect of anticoagulant type, transportation time, and the addition of glucose to the culture medium on the viability and *in vitro* functionality of blood derived PMN of nulliparous, clinically healthy Holstein heifers. Our results indicate that the choice of anticoagulant (ACD or EDTA) had a negligible effect on PMN viability. However, the viability of PMN was negatively impacted by their storage for more than 3 h. Interestingly, the PC capacity of PMN increased at 3 h of storage when compared to other storage time-points. The presence of glucose in the culture medium led to a substantial increase in the *in vitro* PMN OB capacity within 6 h of storage. These findings provide valuable insights into the standardization of PMN storage time and *in vitro* functionality evaluation protocols.

Our findings indicate that the type of anticoagulant, ACD versus EDTA, did not affect the proportion of viable PMN and had a minimal impact on their *in vitro* assessment of PC and OB capabilities at any time point of storage (from 0 to 12 h). These results are consistent with research carried out on human-derived blood PMN by Krabbe et al. (2020) [19]. They reported that anticoagulants (sodium citrate and EDTA) used during blood collection had minimal effects on *in vitro* PMN OB capacity. Mechanism of action of most anticoagulants, including ACD and EDTA, work by chelating calcium ions, forming non-ionised calcium [20]. Thus, as sodium citrate and ACD use an identical mechanism to prevent coagulation, this can explain the lack of differences between anticoagulants shown by our results. However, it was described that EDTA may cause pseudo-neutropenia in blood samples as it makes PMN to clump *in vitro* [21], which can interfere with the PMN isolation process. We did not find differences in reaching the 10,000 events in the flow cytometry at any time-point of incubation in ACD or EDTA isolated PMN. As hypothesized initially, the presence of glucose in the ACD tubes did not influence the longevity nor *in vitro* functions of PMN. Our speculation centers around two key factors: first, the storage conditions at 4°C may have contributed to reduced cellular metabolism, and secondly, PMN were isolated from the enriched glucose environment before conducting the *in vitro* functionality tests.

The viability of PMN remained stable during the first 3 h of storage, then it decreased to less than 80% of viable PMN at 6 h of storage and further. Correspondingly, the proportion of apoptotic and necrotic PMN followed the opposite trend from 6 h onwards. Thus, our results show that after blood collection and storage at 4°C, blood PMN should be isolated within 3 h to maintain high viability. It is important to note that haemoconcentration (sedimentation of formed blood elements) likely occurred in samples stored for >3 h, contributing to the lower PMN viability observed at this time point and beyond. Interestingly, other studies have reported storage of PMN at RT and 37°C [22–24]. However, it was previously reported that storage at 4°C is more efficient in keeping PMN counts, viability, and functionality stable over time when compared to RT and 37°C [19]. Thus, anticipating potential issues related to haemoconcentration, we decided to store our samples at 4°C to slow down cellular metabolic processes. Importantly, before processing, all samples were gently mixed, and none showed visible blood clots on the walls of the collection tubes. Furthermore, based on personal observations, it is worth mentioning that the changes in temperature during storage is likely to activate PMN (e.g., *in vitro* cell aggregation in samples in direct contact with ice during transportation), emphasizing the importance of maintaining a stable temperature during transportation and subsequent PMN isolation.

Interestingly, when combining the data of ACD and EDTA coated tubes, PC increased from 0 to 3 h, reaching a peak at this last time-point. Then, it progressively declined at 6, 9, and 12 h of storage irrespectively of the presence of glucose in the culture medium. It was described that circulating PMN primarily rely on glucose for their metabolism and function, and then progressively use their glycogen stores as a source of energy [25]. We hypothesize that viable PMN are capable of effectively utilizing glucose from their environment (or glycogen from their stores) to reach their PC apogee after 3 h of storage. Then, their PC capacity started to drop either due to their decline in viability, reduction of the glucose concentration in the environment, or depletion in the PMN glycogen stores. It is important to mention a limitation of the PC capacity methods described in the present paper. The PC measurement does not explicitly differentiate between the adherence of fluorescent beads to PMN (which become sticky during activation) and the actual ingestion of these beads. This may have slightly affected the measured PC capacity of PMNs. However, in a previous study, Pascottini et al. [26] found a significant positive correlation between PC and the intracellular proteolytic degradation of fluorescently labeled ovalbumin. This indicates that most of the fluorescent beads identified by flow cytometry are indeed ingested by PMN.

Garcia and colleagues [18] have shown that supplementation of glucose into PMN (isolated from blood of early and mid-lactation cows) culture medium mildly increased PC and OB capacities *in vitro*. Nevertheless, it is crucial to highlight that the baseline glucose concentration in the culture medium employed in their study was 7.2 mM. While in the treatment group, an additional supplementation of 4 mM glucose was done, resulting in a total glucose concentration of 11.2 mM. The blood glucose concentrations in early and mid-lactation cows are around 2.6 and 3.2 mM, respectively [27]. Hence, the glucose supplementations conducted in the study by Garcia et al [18] markedly surpassed physiological standards in cattle. Consequently, their findings should be approached with careful consideration and interpretation.

In the present study, we observed a boost in the OB activity when glucose was supplemented into the cell culture medium in comparison to no glucose supplementation. This observation partially agrees with *in vivo* findings where circulating PMN have fewer glycogen stores and lower OB capacity at calving [25]. However, glycogen stores are not the same as glucose in the extracellular space, since PMN require a constant supply of immediately available glucose to support their function [28]. Specifically, glucose acts as a fundamental substrate for PMN generation of reactive oxygen species (which are notably increased during the OB process) and facilitates endocytosis [29], highlighting the importance of glucose availability for optimal PMN function. It is worth noting that the majority of commercially available cell culture media are formulated with supraphysiological levels of glucose. These formulations are primarily designed for human experiments, considering that healthy human blood glucose concentrations (ranging from 3.9 to 5.6 mM) are higher than those observed in cows. Therefore, supplementation towards ‘human standards’ of glucose concentrations to the culture medium of PMN may artificially increase their OB capacity and could potentially mask impaired PMN function *in vivo*. We speculate that this observation is particularly important in the context of evaluating PMN function in transition cows, when blood glucose concentrations are predominantly lower than in other physiological stages. As mentioned earlier, PC was not affected by the addition of glucose into the PMN culture medium [30], suggesting that PMN do not rely on glucose uptake from extracellular space for this specific function. Our results emphasize the need for further investigation into the role of glucose on different aspects of PMN functionality.

## Conclusions

The results of the present study show that: 1) the anticoagulant (ACD or EDTA) used to collect blood samples does not have major effects on PMN viability and *in vitro* functionality; 2) the storage of blood for more than 3 h at 4°C significantly impairs PMN viability, and 3) 7.2 mM glucose, a common concentration used in the formulation of cell culture media, drastically increases *in vitro* OB capacity of PMN, artificially increasing actual PMN function and potentially masking significant biological impairments. These findings highlight the importance of optimizing experimental conditions to obtain reliable and stable results when studying PMN functionality in dairy cattle.

## Supporting information

S1 Table Fluorescent dyes and their excitation and emission characteristics used for the flow cytometric analyses. (DOCX)
