# Supplementary material for: Effect of type of anticoagulant, transportation time, and glucose in the culture media on neutrophil viability and function test results in dairy cattle
Source: PLoS One. 2024 Oct 10;19(10):e0311742. doi: 10.1371/journal.pone.0311742 (PMC11466419; doi:10.1371/journal.pone.0311742)
Supplement: S1 Table — (DOCX) [file pone.0311742.s001.docx]

**Effect of type of anticoagulant, transportation time, and glucose in the culture media on neutrophil viability and function test results in dairy cattle**

Sanjana Malledevarahalli Chandrappa^1,2,¶^, Lei Xie^1,¶,*^, Sebastian Gonzalez Andueza^1^, Hafez Sadeghi^1,3^, Muhammad Hussnain Rashid^1^, Mehrnaz Niazi^1^, Kaixi Qiao^4^, Qiang ^Dong1,4^, Leila Vincenti^2^, Alessandro Ricci^2,&^, Osvaldo Bogado Pascottini^1,5,&^ , Geert Opsomer^1,&^

*^1^Department of Internal Medicine, Reproduction and Population Medicine, Faculty of Veterinary Medicine, Ghent University, Merelbeke, 9820, Ghent, Belgium*

*^2^Department of Veterinary Sciences, University of Turin, Largo Paolo Braccini 2, Grugliasco, 10095, Turin, Italy*

*^3^College of Veterinary Medicine, Oklahoma State University, Stillwater, OK, United States*

*^4^College of Veterinary Medicine, Northwest A&F University, Yangling, Shaanxi, 712100, China*

*^5^School of Veterinary Medicine, University College Dublin, Belfield, Dublin 4, Ireland*

^*^Corresponding author: [lei.xie@ugent.be](mailto:lei.xie@ugent.be)

^¶^Sanjana Malledevarahalli Chandrappa and Lei Xie should be considered as joint first authors

^&^Alessandro Ricci, Osvaldo Bogado Pascottini, and Geert Opsomer should be considered as joint last authors

**Supplementary Table S1.** Fluorescent dyes and their excitation and emission characteristics used for the flow cytometric analyses.

| **Fluorescent dye** | **Channel** | **Excitation (nm)** | **Emission (nm)** | **Flow cytometric parameter** |
| --- | --- | --- | --- | --- |
| Alexa Fluor 647 | APC | 638 | 660/20 | Viability assessment (secondary antibody) |
| Annexin-V-Fluos | FITC | 488 | 525/40 | Viability assessment (Apoptosis) |
| Propidium Iodide | PE | 488 | 585/42 | Viability assessment (Necrosis) |
| H2DCFDA | FITC | 488 | 525/40 | Oxidative Burst |
| FluoSpheres yellow green | FITC | 488 | 525/40 | Phagocytosis |
